# Evaluating the equity impact and cost-effectiveness of digital adherence technologies with differentiated care to support tuberculosis treatment adherence in Ethiopia: protocol and analysis plan for the health economics component of a cluster randomised trial

**DOI:** 10.1186/s13063-023-07289-x

**Published:** 2023-04-24

**Authors:** Nicola Foster, Amare W. Tadesse, Christopher Finn McQuaid, Lara Gosce, Tofik Abdurhman, Demelash Assefa, Ahmed Bedru, Rein M. G. J. Houben, Kristian van Kalmthout, Taye Letta, Zemedu Mohammed, Job van Rest, Demekech G. Umeta, Gedion T. Weldemichael, Hiwot Yazew, Degu Jerene, Matthew Quaife, Katherine L. Fielding

**Affiliations:** 1 Department of Infectious Disease Epidemiology, London School of Hygiene & Tropical Medicine, London, UK; 2 KNCV Tuberculosis Foundation, Addis Ababa, Ethiopia; 3 KNCV Tuberculosis Foundation, The Hague, The Netherlands; 4 National Tuberculosis Control Program, Ethiopian Ministry of Health, Addis Ababa, Ethiopia; 5 School of Public Health, University of Witwatersrand, Johannesburg, South Africa

**Keywords:** Digital adherence technology, Tuberculosis, Treatment adherence, Equity, Ethiopia

## Abstract

**Background:**

Tuberculosis remains a leading infectious cause of death in resource-limited settings. Effective treatment is the cornerstone of tuberculosis control, reducing mortality, recurrence and transmission. Supporting treatment adherence through facility-based observations of medication taking can be costly to providers and patients. Digital adherence technologies (DATs) may facilitate treatment monitoring and differentiated care. The ASCENT-Ethiopia study is a three-arm cluster randomised trial assessing two DATs with differentiated care for supporting tuberculosis treatment adherence in Ethiopia. This study is part of the ASCENT consortium, assessing DATs in South Africa, the Philippines, Ukraine, Tanzania and Ethiopia. The aim of this study is to determine the costs, cost-effectiveness and equity impact of implementing DATs in Ethiopia.

**Methods and design:**

A total of 78 health facilities have been randomised (1:1:1) into one of two intervention arms or a standard-of-care arm. Approximately 50 participants from each health facility will be enrolled on the trial. Participants in facilities randomised to the intervention arms are offered a DAT linked to the ASCENT adherence platform for daily adherence monitoring and differentiated response for those who have missed doses. Participants at standard-of-care facilities receive routine care. Treatment outcomes and resource utilisation will be measured for each participant. The primary effectiveness outcome is a composite index of unfavourable end-of-treatment outcomes (lost to follow-up, death or treatment failure) or treatment recurrence within 6 months of end-of-treatment. For the cost-effectiveness analysis, end-of-treatment outcomes will be used to estimate disability-adjusted life years (DALYs) averted. Provider and patient cost data will be collected from a subsample of 5 health facilities per study arm, 10 participants per facility (*n* = 150). We will conduct a societal cost-effectiveness analysis using Bayesian hierarchical models that account for the individual-level correlation between costs and outcomes as well as intra-cluster correlation. An equity impact analysis will be conducted to summarise equity efficiency trade-offs.

**Discussion:**

Trial enrolment is ongoing. This paper follows the published trial protocol and describes the protocol and analysis plan for the health economics work package of the ASCENT-Ethiopia trial. This analysis will generate economic evidence to inform the implementation of DATs in Ethiopia and globally.

**Trial registration:**

Pan African Clinical Trial Registry (PACTR) PACTR202008776694999. Registered on 11 August 2020, https://pactr.samrc.ac.za/TrialDisplay.aspx?TrialID=12241.

**Supplementary Information:**

The online version contains supplementary material available at 10.1186/s13063-023-07289-x.

## Introduction

Tuberculosis remains a leading infectious cause of death, disproportionately affecting people living in low-resource settings [1]. Ineffective treatment leads to increased mortality, recurrence, medication resistance, transmission and risk of poverty [2]. Several strategies have been recommended to improve the effectiveness of treatment outcomes.

Historically, tuberculosis programmes emphasised directly observed treatment short-course strategy (DOTS) in high-burden countries [3]. The core of this strategy was the observation of patients by healthcare workers when they take medication. However, this approach is time intensive for patients and providers and became a role performed by peer supporters [4]. While the evidence is mixed, there is some evidence that DOT is no more effective than self-administered treatment (SAT) with adherence support [5–8], but there is improved perceived patient autonomy and reduced healthcare worker costs with SAT [9, 10]. More recently the World Health Organization (WHO) included people-centred tuberculosis care as one of the pillars of the End-TB strategy [11]. Digital adherence technologies (DATs) offer an approach for treatment adherence support by measuring daily doses and monitoring non-adherence with minimal visits to health facilities [4]. Technologies proposed include patient devices such as digital pill boxes, phone applications, platforms for visualising dosing histories, data servers to support video communication, medication envelopes and ingestible sensors [4]. Evidence of the components of these interventions that improves the effectiveness and cost-effectiveness of DATs is limited [8].

In Uganda, a step-wedged trial of medication sleeves, where patients call a toll-free number revealed when a medication dose is removed from the blister pack (99DOTS), found no evidence of improved treatment success during the intervention period [12]. This study estimated that 99 DOTS cost US$303 (range: US$220; US$2119) per person [13]. A cohort study of tuberculosis treatment adherence in Peru found that 8% of the general population and 18% of patients with tuberculosis had no access to mobile phones and found an association between poor treatment outcomes and no mobile phone access [14]. There is therefore a need for further evidence to understand whether the benefit of implementing DATs is fully realised in poor households. We aim to address this evidence gap by examining the cost-effectiveness, and equity impact of the implementation of DATs followed by differentiated care. The ASCENT consortium is evaluating the implementation of two DATs, the digital pillbox EvriMed and medication labels, in five countries: Ethiopia, Tanzania, Philippines, South Africa and Ukraine. The focus of this paper is the protocol and analysis plan for the health economics component of the ASCENT-Ethiopia trial, a cluster randomised trial in Ethiopia [15, 16].

The objective of this study is to estimate the cost-effectiveness of the interventions compared to the standard of care. The secondary objective of the study is to estimate the distribution of the costs and outcomes of the intervention by household socio-economic position. The objectives of the trial are to evaluate whether the implementation of (i) digital pillboxes or (ii) medication labels with daily monitoring using a web-based platform, followed by a tailored response by healthcare workers to adherence data, decreases the proportion of adult pulmonary drug-susceptible tuberculosis patients with unfavourable outcomes compared to the standard-of-care. The primary trial outcome will be assessed as a composite outcome of poor end-of-treatment outcomes (treatment failure, death, lost to follow-up) or recurrence of tuberculosis disease within six months of the end of treatment. Secondary objectives include to (a) describe longitudinal technology engagement, (b) describe the fidelity to the adherence tools, (c) project the epidemiological impact of intervention scale-up and (d) estimate the cost-effectiveness of the intervention compared to the standard-of-care.

## Methods

### The ASCENT-Ethiopia trial

We are conducting a three-arm pragmatic cluster randomised trial where the unit of randomisation is health facilities [15]. A total of 78 health facilities (26 per study arm) in the Addis Ababa and Oromia regions of Ethiopia are participating in the trial. Selected facilities were randomised (1:1:1) using stratified restricted randomisation to provide either (i) digital pillboxes called EvriMed, (ii) medication labels or (iii) standard-of-care. Health facilities were selected based on region, sampling from urban and rural facilities and having at least 30 adult pulmonary drug-sensitive tuberculosis (DS-TB) notifications in 2018.

We plan to enrol 3900 patients (≥ 18 years old) with pulmonary DS-TB in the trial (50 per facility) over a 12-month period and follow them up 12 months after treatment initiation. Participants with bacteriologically confirmed tuberculosis at enrolment, a successful end of treatment outcome and able to produce sputum, will provide a sputum sample for culture approximately 6 months after the end of treatment to measure disease recurrence among trial participants. Primary study outcome is a composite unfavourable outcome of (i) treatment lost-to-follow-up, death, and treatment failure and (ii) disease recurrence within 6 months of end of treatment.

### Intervention

**Fig. 1:**
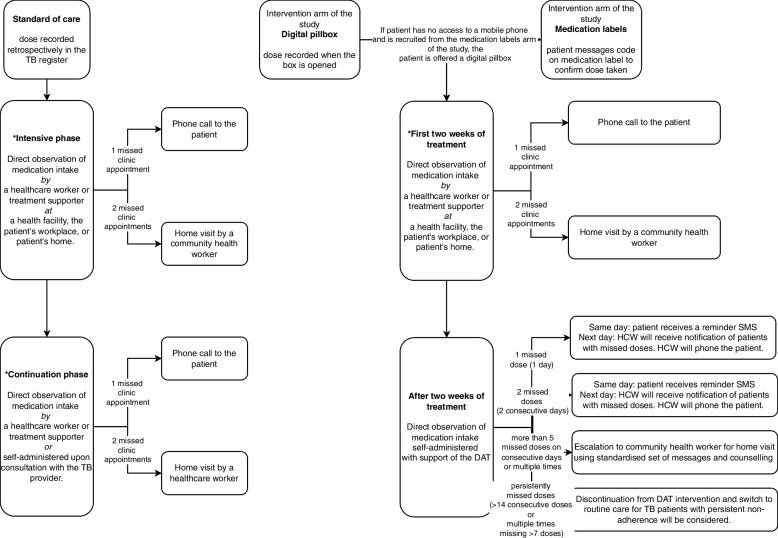
Patient flow through the standard of care and intervention arms of the study *The number of clinic visits has changed during the COVID pandemic. In practice, patients are provided with a 1- to 2-week supply of medication at a time unless the healthcare workers’ assessment signals adherence as a potential concern. TB, tuberculosis; DAT, digital adherence technologies; HCWs, healthcare workers

The intervention is DATs, the digital pillbox called EvriMed or medication labels linked to a data platform and differentiated care based on adherence data. Each dose taken by patients is logged on the platform either automatically when the digital pillbox is opened, or when the patient texts a code on the dose label to a dedicated toll-free number. Healthcare workers have access to the ASCENT data platform on a web-based platform or mobile phone app, which allows treatment adherence to be evaluated and support updated. A flow diagram indicating decision points and actions is shown in Fig. 1. Participants recruited from intervention facilities are offered a DAT and 2-weeks’ worth of medication with a return appointment. The intervention in the labels arm of the study requires the use of a mobile phone to text codes indicating that they have taken a dose and a mobile phone is not required. For the digital pillbox, the patient does not require having a phone as doses are logged automatically when the box is opened to take a dose. Patients enrolled in the labels arm of the study who do not have a mobile phone will be offered a digital pillbox.

In the standard-of-care arm, doses are retrospectively recorded in the facility tuberculosis record during treatment visits. Prior to the COVID-19 pandemic, patients in the intensive phase of treatment received DOT. Currently, patients are self-administering medication for 1 to 2 weeks unless the providers’ assessment identifies adherence as a concern. If clinic appointments are not attended, patients will be phoned to provide adherence counselling. If further clinic appointments are missed, a community health worker will conduct a home visit.

In the intervention arms of the study, one missed clinic appointment will be followed up telephonically. Following two consecutive missed clinic visits, a community health worker will visit the home. After the first 2 weeks, for each missed dose, patients receive a same-day automated reminder SMS. If two or more consecutive doses are missed, the patient will receive a telephone call. More than five missed doses result in a home visit and if doses are persistently missed (> 14 consecutive doses or multiple times missing 7 doses), discontinuation from the DAT intervention and switch to daily DOT under healthcare workers’ observation will be considered.

Possible harms due to study participation include the risk of inadvertent disclosure of patients’ tuberculosis status because of the use of digital pillboxes or receiving SMS notifications. These harms are monitored by keeping a health facility social harm register. Inadvertent disclosures are recorded at the 12-month follow-up and reviewed by the ASCENT Trial Advisory Group [15].

### Population

Adults (≥ 18 years old) receiving pulmonary DS-TB treatment at the trial facilities are screened for eligibility by facility healthcare workers, and informed consent is requested from eligible patients. Participant exclusion criteria include patients who are expected to migrate out of the health facilities’ catchment area in the next 12 months and patients receiving in-patient or palliative care. Participants who withdraw from the study or stop using a DAT will continue to receive routine care and asked if their adherence and treatment outcome data may be collected.

The primary analysis will be an intention-to-treat (ITT) analysis, where the costs and effects of all participants enrolled in the trial are included, excluding participants whose diagnosis are changed to ‘no TB’, are started on MDR treatment, or are transferred out ≤ 28 days of treatment start. As a sensitivity analysis, we will conduct a per-protocol (PP) analysis that excludes participants in the intervention arm who opted out of using DAT during the treatment period, started DAT ≥ 28 days after treatment initiation, was withdrawn from using DAT due to poor network, lost phone or poor adherence.

### Sample size

Sample size estimation for the primary trial outcomes considered the clustered study design and pair-wise comparisons of the standard-of-care—to the intervention arms [15]. Programmatic tuberculosis treatment outcome data from trial health facilities’ poor end-of-treatment outcomes were recorded for 17% of patients. For the trial, we include an additional unfavourable treatment outcome, disease recurrence 6 months after end-of-treatment. Assuming that between 17 and 20% of patient experience unfavourable outcomes, a harmonic mean of 50 adult tuberculosis registrations per facility, 26 facilities per study arm and a coefficient of variation of the outcome between 0.25 and 0.35, the trial will have 80–85% power to detect a one-third reduction in an unfavourable outcome.

### Timelines

Data collection for the study is expected to be completed by June 2023, and the results of the trial analyses available in October 2023. There are no interim analyses planned.

### Data-generating processes

Treatment outcomes and events will be collected for 3900 trial participants. Estimates of provider and patient costs will be collected from a sub-sample of 15 health facilities, 5 facilities per study arm and 10 participants per facility, preserving the empirical correlation between effects and costs and therefore providing a more accurate estimate of the uncertainty interval of the average cost-effectiveness estimate [17, 18]. Health facilities were selected for the sub-sample based on study arm, region (Addis or Oromia), urban versus rural and regional poverty rate. Participants were selected based on their gender and time-on-treatment. Data sources and data collection methods are described in Table 1.

**Table 1:** Data generating processes by data source and indicator

**Data source**	**Description of data**	**Used to estimate**
Tuberculosis treatment register	For all participants enrolled in the trial, baseline data are collected from the facility tuberculosis treatment register, including gender, age, HIV and ART status and treatment history From this register, the following outcomes will be abstracted, including cured, treatment completed, treatment failure, death, loss to follow-up, not evaluated and moved to the MDR register. The following patient resource-use data collected (1) number of tuberculosis diagnostics and (2) number of months on tuberculosis treatment	Resource use Treatment outcomes
Baseline socio-demographic survey	All participants enrolled on the trial are interviewed at enrolment to collect baseline demographic information as well as information to estimate household socio-economic position	Household socio-economic position
12-month follow-up survey	Participants enrolled on the trial with successful (completed/cure) end-of-treatment outcomes will be interviewed 12 months after enrolment to confirm their treatment outcomes and to ask additional questions about their resource use during the preceding year, including (3) number of hospitalisations and nights in hospital, (4) number of adherence support home visits and (5) treatment restarted	Resource use
Health facility visits log	Completed by healthcare workers in the trial facility and (6) number of visits made to a trial health facility	Resource use
Substudy 1 patient survey	This survey will be implemented in a subsample of 15 (of 78) health facilities. In each health facility, approximately 10 trial participants will be recruited to complete a survey on patient costs incurred. Purposive sampling will be used to sample participants who are in the intensive versus continuation phase of treatment and to achieve a balanced sample of men and women. Patient resource use data that will be collected include (7) number of visits to a private health facility and (8) number of phone calls related to adherence	Patient unit costs Resource use
Facility surveys A and B	HCWs from a subsample of 15 (of 78) health facilities, will be asked to collect using the facility costing data related to the cost of healthcare worker time collection tool. The time spent by healthcare workers on activities related to treatment adherence will be collected using two facility surveys: Survey 1: healthcare workers will be asked to, over a period of 6 weeks, record the time they spend on treatment support-related tasks during interactions with 10 consecutive participants Survey 2: asked to retrospectively record time spent on administrative tasks weekly for 6 weeks	Provider unit costs
Cost data collection tool	Used to collect data on facility overhead costs (such as building space, maintenance, utilities and management staff). These costs will be allocated to the tuberculosis programme and to the individual patient level using the proportion of human resource time as the allocation factor	Overhead costs
Visits and calls ASCENT-platform	Project staff logs their interactions with health facility staff (visits and calls) in supporting the implementation of DATs and responding to queries related to the ASCENT adherence platform	Intervention costs
KNCV project records	Training logs and project expenditure reports of the cost of procuring and distributing DATs will be collected	Intervention costs

Further detail is provided in Additional file 1: S1 Text

Baseline participant demographics and household socio-economic position (SEP) will be collected at study entry for all trial participants. Enrolled participants’ treatment outcomes (cured, treatment completed, treatment failure, death, loss-to-follow-up, not evaluated and MDR-TB) will be abstracted from the facility tuberculosis treatment register by health facility staff. Twelve months after treatment initiation, trial participants with a successful end-of-treatment outcome will be contacted to confirm their outcomes. Health facility visits logs completed by healthcare workers at the subsample health facilities will be used to record all visits made by trial participants. Direct- and indirect patient unit costs will be collected from ten participants per facility (*n* = 150), in the intensive or continuation phase of treatment. Provider unit costs will be collected from subsample facilities using two facility surveys (facility surveys A and B) completed by the facility TB focal person over a period of 6 weeks. Surveys will be used to record time spent on tuberculosis treatment-related tasks with ten consecutive participants and the amount of time spent weekly on adherence support (including home visits). A facility cost data collection tool will be used to collect provider cost data. The cost of implementing the intervention will be estimated using KNCV records on the cost of purchasing and distributing DATs. The cost of training and supporting healthcare workers in implementing DATs will be estimated using KNCV training logs and staff interactions with health facilities logged on the ASCENT platform.

### Analysis

**Fig. 2:**
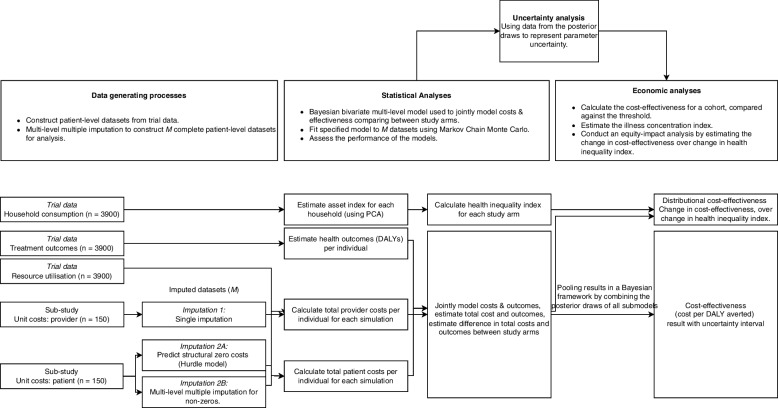
Schematic of the data-generating processes and analyses

We will conduct a within-trial cost-effectiveness analysis using patient-level data from a cluster randomised trial and therefore need to consider the following in the choice of analytical approaches (1) costs and effectiveness endpoints will be correlated, (2) data will be hierarchical because of the randomisation of facility clusters in the trial whereby each cluster is distinct from the other clusters and data within clusters are broadly similar, (3) cost data tend to be right skewed and therefore model assumptions of normally distributed data will not hold, (4) structural zero costs where some data points are expected to be zero, and (5) partially observed data because costs are collected from a subsample of facilities [19, 20]. We will jointly model costs and a continuous effectiveness measure, disability-adjusted life years (DALYs), using Bayesian hierarchical models [21, 22]. Thereafter, we will estimate the distribution of costs and effects by household socio-economic position using cost and illness concentration indices [23]. The links between the data generation processes and analyses are shown in Fig. 2, and further detail is provided in Additional file 1: S1 Text. Analyses will be conducted using R and STATA 17 [24, 25].

Baseline participant characteristics that will be summarised and compared between study arms are shown in Table 2. Important differences in baseline characteristics will be adjusted for and both an adjusted and unadjusted analysis result presented [26].

**Table 2:** Baseline participant characteristics to be reported by the study arm

**Characteristic**	**Presented**	**Measure**
Age	Continuous	Years
Gender	Categorical	Male Female
Household socio-economic position (SEP)	Ordinal	Poorest Less poor Middle More wealthy Wealthiest
Facility region	Categorical	Addis Ababa Oromia
Tuberculosis disease classification	Categorical	Bacteriologically confirmed Clinically diagnosed
Treatment history	Categorical	New Relapse Treatment after failure Treatment after loss-to-follow-up Others previously treated Previous treatment unknown
HIV status	Categorical	Positive Negative Unknown
ART status	Categorical	On ART Not on ART Unknown
Educational level	Categorical	Not attended school No formal education but can read and write Primary school not completed Primary school Secondary school Post-secondary certificate University level courses Attended religious school Others
Number of people in the household	Categorical	1–4 5–8 > 8
Marital status	Categorical	Unmarried living with parents Unmarried living alone Cohabiting Married, living with a spouse Married, not living with a spouse Separated Others
Frequency of income received	Categorical	Monthly/weekly Seasonally Once in a while No income

*HIV* Human immunodeficiency virus, *ART* Antiretroviral status, *DAT* Digital adherence technologies

### Cost estimation and imputation

We will use an ingredient costing approach to calculate the provider cost of human resources, training, and implementing the intervention in 15 of the 78 trial facilities [27]. Overhead costs will be allocated to the intervention in a top-down manner using health service utilisation as the allocation factor. Medication costs will be included based on number of months of medication dispensed. Single imputation will be used to impute unit costs from the sub-sample facilities to all patients based on the facility (study arm, area and patient time on treatment) they received care.

Patient costs include the direct costs, the indirect costs, and the value of assets sold due to hardship experienced due to illness or accessing treatment. Patients’ time will be valued using self-reported monthly income and country-specific minimum wage in the sensitivity analysis. The total patient cost will be estimated as the sum of direct costs, the value of time lost due to accessing treatment and the value of any assets sold. We will report the number of patients experiencing catastrophic costs as the number of patients for whom total patient costs exceed 10% of their self-reported income [28]. The cost per patient incurred by patients at the last patient visit is collected from a subsample of 150/3900 study participants and then imputed to all participants using multivariate multi-level multiple imputation (see Fig. 2) [29]. Missing unit cost data will be imputed using predictive mean matching, generating 10 (*M*) complete datasets [29].

### Statistical analyses

Statistical models will be used to estimate the differences in costs and outcomes between study arms and to analyse associations between patient-level factors and costs/outcomes. For each of the 10 (*M*) complete datasets, costs and effectiveness will be modelled jointly using Bayesian hierarchical models to account for the clustered nature of the data. Random effects will be specified to allow for individual-level variances that differ across clusters. The cost and effectiveness variables will be modelled using zero-inflated Poisson regression models, therefore not assuming that zero and non-zero values come from the same data-generating processes [20, 30]. Cost data are typically right skewed and by fitting a zero-inflated Poisson model, the cost variable will be modelled as a mixture of a Bernoulli and Poisson distribution reflecting the skewed nature of the data [31]. The models will be fitted using Bayesian inference by obtaining posterior simulations given the model and data, using Markov chain Monte Carlo sampling. The results of the imputed models will be pooled in the Bayesian framework by combining the posterior draws of all *M* submodels [32].

### Cost-effectiveness analysis

Total costs incurred per patient are estimated by multiplying utilisation (such as number of clinic visits) by a unit cost (for example, provider cost per visit) [33]. The valuation approaches used and outcomes assessed are summarised in Table 3. The perspective of the analysis will be societal and costs of both the provider and patient included in the analysis. The analytical time horizon will be from the start of tuberculosis treatment to the end of the current treatment episode. If a trial participant has a 12-month outcome recorded as recurrence or an MDR-TB diagnosis, the costs and outcomes associated with MDR-TB treatment and additional courses of treatment will be included. Overhead costs will be allocated to the TB programme and to a per-patient cost using the proportion of human resource time used as an allocation factor. We will discount costs and benefits occurring in the future, using a 3% discount rate and varied between 0 and 10% in the sensitivity analyses [33].

**Table 3:** Valuation approach and outcomes estimated

**Analysis**	**Valuation**	**Outcomes**
Societal costs	Patient costs: the sum of all out-of-pocket costs incurred by patients in accessing treatment and treatment support, plus the cost of time spent in accessing treatment. The proportion of patients who incur catastrophic costs is estimated by the number of patients who incur costs that exceed 10% of their self-reported income, as a percentage of all TB patients Provider costs: the sum of costs incurred by the health service in providing care. An ingredient costing approach is used to identify the costs of purchasing and implementing the technology, plus the human resource costs to provide treatment support. Overhead costs incurred by the health facility are allocated down to the intervention using an allocation factor based on facility workload data. Societal costs refer to the sum of patient-incurred and provider costs Poverty case: a participant will be counted as a poverty case if the direct costs incurred (e.g. out-of-pocket costs of transport to clinics and purchasing healthcare) reduce their self-reported income to below the national poverty line in Ethiopia	Patient cost per patient Provider cost per patient Societal cost per patient Proportion of patients who incurred catastrophic costs Poverty case
Disability-adjusted life years	Disability-adjusted life years (DALYs) is an outcome measure that is the sum of years of life lost due to premature mortality (life expectancy in country minus age at death) and the years of life lived with the disease where the number of years lived with the disease is adjusted by a utility weight. Mortality/deaths as recorded in the treatment register and/or during the 12-month follow-up will be used to estimate years of life lost. Years of life lived with a disability (YLD) will be the time from diagnosis to successful treatment estimated from the trial outcomes	Years of life lost (YLL) Years of life lived with a disability (YLD) Disability-adjusted life years (DALYs)
Cost-effectiveness	Cost-effectiveness is estimated as the difference in the per-patient costs incurred in the intervention compared to the standard of care arm of the study, divided by the difference in DALYs between the two study arms. If the cost per DALY averted value (incremental cost-effectiveness ratio) for the intervention is below the cost-effectiveness decision threshold, the intervention will be considered cost-effective	Cost per unfavourable outcome averted Cost per DALY averted
Equity impact analysis	The distribution of costs and outcomes by household socio-economic position (SEP) will be assessed by estimating the illness—and cost-concentration indices by study arm. The illness concentration index is defined as twice the area between the concentration curve and the line of equality on a graph of the cumulative percentage of people in the same ranked by economic status against the cumulative percentage of ill health	Distribution of costs and outcomes by household SEP Illness concentration index (ICI) for each study arm Cost concentration index (CCI) for each study arm

*TB* Tuberculosis, *SEP* Socio-economic position, *DALY* Disability-adjusted life years, *YLL* Years of life lost, *YLD* Years of life lived with a disability, *ICI* Illness concentration index, *CCI* Cost concentration index

The mean differences in costs and DALYs with be estimated through pair-wise comparison of each study arm to the standard-of-care study arm, and cost per DALY averted ranked from the lowest to highest number. An intervention will be considered cost-effective if (i) less costly and more effective than the standard-of-care or (ii) more costly and more effective but the incremental cost-effectiveness ratio (ICER) is below the cost-effectiveness threshold (CET).

CETs are country- and payer-specific, representing the opportunity costs of investing in alternative health interventions [34–36]. We will use a range of CET estimates, based on Ochalek et al.’s empirical estimates of CETs using estimates of the mortality effect of health expenditure, between US$8 and US$9 and the results of a review of the Ethiopian essential health benefit package that suggests a threshold of US$2000 per DALY averted would be acceptable [34, 37, 38]. Equity is one of the seven prioritisation criteria used in the Ethiopian Essential Health Service Package and we will therefore present an equity-efficiency trade-off analysis in addition to a traditional cost-effectiveness analysis [39, 40].

### Equity and distributional cost-effectiveness analysis

An asset index representing household SEP will be created using principal components analysis (PCA) of 23 variables measuring various dimensions of poverty. The questions are based on previous studies conducted in Ethiopia and include (i) education, (ii) frequency of household income, (iii) marital status, (iv) access to sanitation, (v) cooking fuel, (vi) household crowding and (vii) ownership of consumer goods, land and livestock [41–45]. The asset index will be used as a continuous variable and grouped into terciles to rank households from poor to wealthy.

Traditional cost-effectiveness analyses implicitly assume that costs and outcomes are distributed equally within the cohort irrespective of household socio-economic position (SEP). Here, we extend the decision framework to include measures of the distribution of the costs and intervention effectiveness by household SEP, by estimating the cost and illness concentration indices [46, 47]. The concentration index is estimated from a plot of the cumulative proportion of costs/outcomes against the cumulative proportion of the population ranked by SEP beginning with the least advantaged [23, 48, 49]. The concentration index is the area between the concentration curve and the line of equality [50, 51]. If we find an unequal distribution we will use an equity weighting to show the additional benefit of interventions that preferentially benefits households of poorer SEP [47, 52].

### Subgroup, sensitivity and uncertainty analyses

Subgroup analyses will be conducted to investigate how the intervention effect on outcomes and costs changes in subgroups defined by SEP, gender, time on treatment, health service utilisation and geographic region. Planned sensitivity analyses are summarised in Table 4, and include per-protocol analyses, examining the impact of changes in the frequency of health facility visits, varying discount rates, varying the catastrophic costs threshold and the cost of DATs. Additional sensitivity analyses conducted will be described as post hoc analyses.

**Table 4:** Sensitivity analyses

**Sensitivity analysis**	**Description of approach**	**Change in outcomes evaluated**
Per protocol analysis	Exclude participants who did not use the DATs to the end of the treatment period	Cost-effectiveness
Changes in the number of healthcare visits	Change the number of healthcare visits to resemble what was observed prior to the COVID epidemic to see the impact on costs and cost-effectiveness	Provider and patient costs
Cost of the DATs	Increase and decrease the cost per person of DATs	Provider costs; cost-effectiveness
Analytical timeframe	Only include the costs and outcomes incurred during the treatment of trial participants until the end of the trial	Cost-effectiveness
Catastrophic costs threshold	Varying the catastrophic costs threshold from between 10 and 20%	Patient costs; cost-effectiveness
Discount rates	Vary the discount rate used for costs and effects from 3 to 0% and 10%	Costs, effects and cost-effectiveness

*DATs* Digital adherence technologies

The MCMC output from the Bayesian model will be used for post-estimation analysis of the parameter uncertainty in the decision analysis. We will estimate whether the intervention(s) would be cost-effective against the standard-of-care at a range of CETs. The results of this analysis will be presented on a cost-effectiveness acceptability frontier, showing the probability that a simulation will be cost-effective for each intervention against a range of CETs [53].

## Discussion

The analysis described here is a within-trial cost-effectiveness—and equity impact analysis evaluating the distributional cost-effectiveness of two digital adherence technologies (DATs) for support of tuberculosis treatment adherence. Both DATs collect dose-by-dose adherence data which can then inform healthcare workers to intervene and support tuberculosis treatment adherence without the need to wait for clinic visits to address challenged and encourage adherence. To date, few studies have produced estimates of effectiveness, the patient and patient costs incurred for dose-by-dose adherence support interventions using randomised studies [8]. Furthermore, there have been concerns raised about the potential inequity in providing support that requires patients to have access to mobile phones [14]. Previous work has shown that adherence to tuberculosis treatment and treatment outcomes are associated with household socio-economic position and that patients from low socio-economic position households have the greatest risk of poor treatment outcomes [54, 55]. We would expect that the implementation of DATs with a structured approach to following individuals up if there are indications of poor treatment adherence would reduce the number of visits required by patients to attend a health facility and associated costs when compared to the standard of care. Conversely, if the number of visits in the standard-of-care arm was lower than we initially considered, influenced by the COVID epidemic or not following the guidelines, then we may see smaller cost differences between study arms. There is currently limited evidence that the implementation of DATs with differentiated care improves the outcomes of high-risk patients, but a lack of evidence of how costs and impact of treatment adherence support are distributed between households of different socio-economic position (SEP), and this may be crucial to our understanding of the value of the implementation of DATs [56]. For example, if DATs reduce patient-associated costs and are more beneficial in reducing the composite score of poor treatment outcomes in households with lower SEP then an argument can be made that implementation should go ahead even if the implementation of the technology is more costly than the standard-of-care. This study will address some of the evidence gap.

This trial was implemented at the time of the COVID pandemic, which resulted in changes in the ways that people access healthcare services in Ethiopia and globally. There is some evidence that routine visits to healthcare facilities have reduced due to the pandemic [57]. What is currently unclear is how changes in treatment guidelines towards fewer DOTS visits, may have changed the comparator for DATs and our assessment of the comparative cost-effectiveness of DATs. Additional unspecified analyses that may therefore be required if the trial analysis shows no difference in the effectiveness of the intervention compared against the standard-of-care, is to investigate what the impact of differences in device uptake and changes to the differentiated care aspect of the intervention has on decisions related to cost-effectiveness of DATs. A limitation of our study is that we consider the costs and effects for the within-trial period only and do not include the cost of onward transmission and the impact on the tuberculosis epidemic in Ethiopia. Further analyses planned as part of the ASCENT consortium are expected to fill this gap. Furthermore, our sample size for the patient costs study is limited with only 150 patients. We therefore cannot empirically assess the difference in costs incurred between rich and poor households; however, by imputing patient costs based on health service utilisation, which is collected for each study participant, we maximise the available sample size.

There are a range of approaches currently used to assess the distributional (equity) impact of national decision-making [46, 47, 58]. Broadly, these analyses can be categorised based on the objective of the estimation: (i) whether burden of ill health differentially falls on households with the fewest resources (disease impact), (ii) whether cost of treatment disproportionally disadvantages the poor (poverty impact) and (iii) whether the decision to invest in each intervention improves the fairness of the distribution of limited resources for healthcare in a country (decision impact). A recent systematic review of equity informative economic evaluations found that these evaluations are most frequently conducted to assess preventative care, with 26% (14/54) evaluating infectious disease interventions. Only 22% (12/54) of the evaluations reported informed decisions on the African continent [46]. Our evaluation will contribute to a growing body of evidence of the equity-informative evaluations of interventions for tuberculosis control [58–60]. We will explore the equity-efficiency trade-offs of investing in DAT compared to other potential investments. The equity trade-offs of interest are whether the distribution of ill health (DALYs) in Ethiopia will change if tuberculosis treatment support is invested in, or whether the cost associated with ill health due to tuberculosis will be distributed differently between households of different SEP because of the investment. The paper presented here, follows on from the trial protocol paper [15], by outlining the protocol and analysis plan for the health economics work package alongside the trial.

## Trial status

Recruitment to the run-in phase of the trial started in December 2020, followed by the main phase in June 2021 and recruitment will continue until June 2022. Follow-up is expected to continue until June 2023. This analysis plan was first drafted at the end of the run-in phase and completed prior to data analysis.

## Supplementary Information

**Additional file 1: S1 Text.**

## Data Availability

Data used in the economic evaluation will be made available through the LSHTM data repository, LSHTM Data Compass. Following the publication of the trial results, there will be a period of exclusive data access for researchers from the ASCENT consortium and local research community in each participating country prior to its availability on LSHTM Data Compass.
