# Supplementary material for: Evaluating the equity impact and cost-effectiveness of digital adherence technologies with differentiated care to support tuberculosis treatment adherence in Ethiopia: protocol and analysis plan for the health economics component of a cluster randomised trial
Source: Trials. 2023 Apr 24;24:292. doi: 10.1186/s13063-023-07289-x (PMC10123464; doi:10.1186/s13063-023-07289-x)
Supplement: Supplementary file 1 — Additional file 1: S1 Text. [file 13063_2023_7289_MOESM1_ESM.docx]

S1 Text

Further technical details to accompany paper “Foster et al. *Evaluating the equity impact and cost-effectiveness of digital adherence technologies with differentiated care to support tuberculosis treatment adherence in Ethiopia: protocol and analysis plan for the health economics work package of a pragmatic cluster randomised trial*”.

Based on the following project documentation:

| ASCENT-Ethiopia protocol | 2.2 (28 February 2022) |
| --- | --- |
| Statistical analysis plan | 0.4 (23 May 2022) |

# **Data generating processes**

We are conducting a “with-in trial” cost-effectiveness analysis. The societal costs and outcomes incurred by trial participants from the time of enrolling into the trial to the end of the 12-month follow-up will be included. Outcome data from 3900 trial participants recruited from 78 health facilities in the Oromia and Addis regions of Ethiopia will be included. Cost data will be collected from a sub-sample of 15 of the 78 trial health facilities. Further detail on how variables will be measured, with reference to survey instruments are described Table S1. Patient-level datasets will be constructed from trial data. The data will be plotted to identify the variables with missing data, and the patterns of missingness in the data (See Figure S1).

Table S1.Measurement and Valuation.

|  |  |  |  | **Sample Size** | | |
| --- | --- | --- | --- | --- | --- | --- |
| **Category** | **Measurement** | **Valuation** | **Data source** | **Nr of clusters** | **Participants per cluster** | **Total sample** |
| Individual-level data | | | | | | |
| Demographics | Age, gender, household characteristics, treatment history, treatment regimen, HIV status. | Used to describe the cohort and as explanatory variables in the analysis. | Sociodemographic case report form | 78 | 50 | 3 900 |
| Household socio-economic position | Household socio-economic position | Principal component analysis to generate a household asset index, based on the consumption and other dimensions of poverty. | Sociodemographic case report form | 78 | 50 | 3 900 |
| Treatment outcomes | Cured, treatment completed, treatment failure, death, loss to follow-up, not evaluated, moved to MDR register. | Treatment outcomes will be assessed as a composite index using data from each patient as recorded in the tuberculosis treatment register. | Health facility tuberculosis treatment register abstraction | 78 | 50 | 3 900 |
| Health outcomes | Disability Adjusted Life Year (DALYs) averted | We will use the data from the observed treatment outcomes to estimate the disability adjusted life years (DALYs) of the trial participants. | Utility data from secondary data sources | 78 | 50 | 3 900 |
| Direct costs | Patient-incurred costs | The out-of-pocket costs incurred by patients in accessing tuberculosis treatment. This is estimated by multiplying the costs incurred by a sample of participants to resource use data collected from all study participants. | Substudy 1 patient data collection form | 15 | 10 | 150 |
| Indirect costs | Time lost by patients due to ill health and accessing healthcare | The amount of time spent by patients traveling to health services, the time they spend waiting to receive healthcare and income loss due to ill health. This data will be used to estimate productivity loss by using (1) occupation data to estimate the possible value of time spent by the individual (2) applying the minimum wage throughout to value the cost of time. | Substudy 1 patient data collection form | 15 | 10 | 150 |
| Resource use | Visits to healthcare services | Counts the number of visits not only to the healthcare facility where they are interviewed but to all healthcare accessed including private healthcare, and pharmacies. | Substudy 1 patient data collection form | 15 | 10 | 150 |
|  | Visit to the public health facility | Number of visits (and reason for visit) of each patient to the health facility. Individual-level data. | Health facility visit log | 78 | 50 | 3 900 |
|  | Telephone calls | Counts the number of treatment adherence related telephone calls made by staff at the health facility to patients in a defined period. | Substudy 1 patient data collection form | 15 | 10 | 150 |
|  | Home visits | Counts the number of home visits made by healthcare workers to the patient’s home or place of work. | 12Month case report form | 78 | 50 | 3 900 |
|  | Time on tuberculosis treatment | Number of months patients spend on treatment at the time of the outcome was recorded. | Health facility tuberculosis treatment register abstraction | 78 | 50 | 3 900 |
|  | Hospitalisation | Counts of time spent in hospital, and the duration of admission. | 12Month case report form | 78 | 50 | 3 900 |
|  | Diagnostic tests | Counts of sputum tests conducted per individual enrolled in the study. | Health facility tuberculosis treatment register abstraction | 78 | 50 | 3 900 |
|  | Diagnostic tests | Chest x-rays and culture tests conducted per individual enrolled in the study | Substudy 1 patient data collection form | 15 | 10 | 150 |
| Uptake | Constraints to the uptake of the interventions. | Measures of constraints to the intervention includes availability of own mobile phone/ smart device; and proportion of devices for which errors are reported which may have limited uptake/ usability. | ASCENT adherence platform data; Substudy1 patient data collection form | 15 | 10 | 150 |
| Facility-level data |  |  |  |  |  |  |
| Direct costs | Overheads (building costs, electricity, water, furniture, and maintenance) | Overhead costs related to the building space and resources used to provide the services. | Will be collected from participating health facilities by contacting the head of the facility. | 15 | - | - |
|  | Human resource costs | The self-reported amount of time spent by healthcare workers on various tasks related to providing tuberculosis treatment and adherence support. Healthcare workers were also asked to report the amount of time they spend on adherence related tasks; and home visits or telephonic follow-ups and administrative tasks. | Substudy 1 facility data collection forms A and B. | 15 | 1 | 15 |
|  | Drug costs | Cost of tuberculosis treatment supplied through the regular pharmaceutical supply chain in Ethiopia. | Secondary data sources | - | - | - |
|  | Hospitalisation | The cost per day to the health service of treating someone in hospital. | Secondary data sources | - | - | - |
| Intervention cost | (Cost of producing DATs) | We will use the cost of producing DATs as a lower bound cost estimate to explore the effect of variation in technology cost in the decision. | KNCV market access costing | - | - | - |
|  | Cost of purchasing DATs | The cost of importing and purchasing the digital adherence technologies, including the MERM box and labels. Includes the costs of any additional training materials. | ASCENT project expenditures | - | - | - |
|  | Training costs | The costs (human resource, accommodation, travel, venue hire & transport) incurred in providing initial training to health care workers in implementing DATs. Time spent on supporting and implementing the research component of the project is excluded from this costing. | ASCENT project training logs | - | - | - |
|  | Support costs | The cost of providing ongoing support for the implementation of the intervention. | Facility communication log: Health facility visits log | - | - | - |
| Resource use | Health facility activity data | Total number of visits made to the health facility enrolled in the study and reason for the visit, this is used as an allocation factor to allocate shared facility level costs to the tuberculosis treatment adherence service. | Health facilities visit log | 78 | All | All |


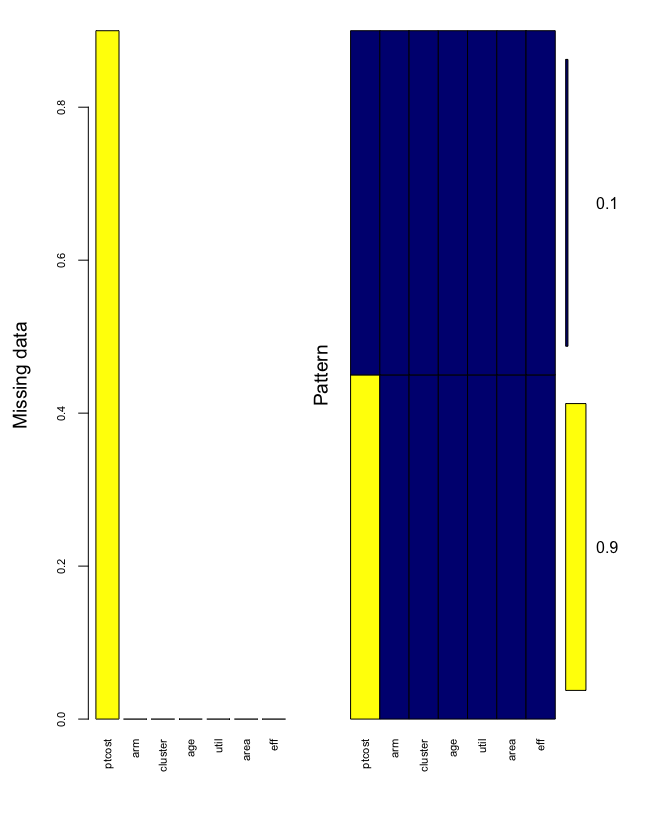


Figure S1. Patterns of missing data in a simulated dataset.

## **Household socioeconomic position**

Household Socio-Economic Position (SEP) will be calculated from the following 23 variables assessed for each household, using Principal Component Analysis (PCA). The variables used to develop the index are listed in Table S2.

Table S2. Variables used to assess household socio-economic position.

| Dichotomous variables, owns the following items |
| --- |
| Owns or rents land |
| Livestock (goat, horse, chicken, cow) |
| Bicycle |
| Car |
| Cart |
| Motorcycle |
| Bed |
| Fridge |
| Lamp |
| Mattress |
| Mitad (large clay disc used to cook injera/ bread) |
| Mobile phone |
| Radio |
| Sofa |
| Television |
| Marital status |
| Ordinal variables |
| Toilet facilities [1=no facility; 2=latrine no slab; 3=latrine with slab; 4=latrine improved; 5=toilet no flush; 6=toilet with flush] |
| Fuel used for cooking [1=wood/ dung; 2=kerosene/ coal/ charcoal; 3=gas/ electricity] |
| Source of drinking water [1=no investment source; 2=piped community/ yard; 3=piped in-house] |
| Income frequency [1=monthly; 2=seasonally; 3=irregularly; 4=no income] |
| House ownership [1=alone only; 2=jointly only; 3=both alone and jointly; 4=does not own] |
| Education |
| Numerical variables |
| Number of people per room |

## **Utilisation and events**

Treatment outcomes and time on treatment will be available per individual enrolled in the trial. The number of clinic visits per patient will be collected using facility logs, with further detail on health service utilization (including the number of visits to private health facilities collected from a sub-sample of 15 of 78 health facilities).

## **Provider unit cost estimation**

Provider costs refer to the costs incurred by the health service provider and may be a public service provider (such as is the case in a tax-funded health system) or a private provider in the case of service provision through a health insurance. The provider costs per visit will be estimated at the facility level for a subsample of 15 of the total 78 health facilities included in the trial. Provider cost per visit will be estimated at the facility level for 15 of 78 facilities for the diagnosis and treatment phases of the treatment episode. Health care worker time use has been empirically collected for a period of 6 weeks. Use single imputation to impute the average cost per health facility to health facilities of similar health service utilization. Intervention and support costs per patient will be estimated at the country level, applied to each patient. Use single imputation to impute the average cost per health facility to health facilities of similar health service utilization.

Provider unit costs will be imputed by single imputation based on the following cluster (facility) characteristics.

Table – number of facilities sampled

| Region | Area | Tuberculosis notifications | |
| --- | --- | --- | --- |
|  |  | High | Low |
| Addis | Rural |  |  |
|  | Urban |  |  |
| Oromia | Rural |  |  |
|  | Urban |  |  |
|  |  |  |  |

Table – number of patients’ data imputed

| Region | Area | Tuberculosis notifications | |
| --- | --- | --- | --- |
|  |  | High | Low |
| Addis | Rural |  |  |
|  | Urban |  |  |
| Oromia | Rural |  |  |
|  | Urban |  |  |
|  |  |  |  |

## **Patient unit cost estimation**

Costs incurred by patients during their last visit to a health facility (whether public, private or hospital) are elicited from patient exit interviews. These surveys will be implemented in 15 health facilities (5 from the control arm and 10 from the intervention arms). Patients will also be asked about the time they spent travelling to and waiting in the health facilities. Costs incurred by patient during the last visit from a health facility elicited from patient exit interviews (15 health facilities, 150 patients imputed for all 78 facilities and 3900 patients). Patient time will be value using individual self-reported income, with minimum wage used as a sensitivity analysis.

For patient unit costs, a multivariate multi-level multiple imputation will be conducted, using the ‘mice’ and ‘brms’ packages in R statistical software. The planned covariate for the imputation will include age, gender, household SEP, facility region and healthcare utilization with a random effect for clustering. Imputation will produce *M* datasets, where *M* will be between 10 and 100 depending on at which point the model converges. Imputation will be by predictive mean marching with 1000 iterations per dataset produced planned. Model convergence will be assessed visually by plotting the imputation chains. Health convergence is when the variance between imputation chains is almost equal to the variance within chains.

The specified statistical model will then be fitted to *M* number of complete imputed datasets within a Bayesian framework with hierarchical modelling with Markov Chain Monte Carlo (MCMC) to obtain posterior simulations given the specified model and data. For this process we will use Stan in R.

## **Health outcomes**

The trial evaluation’s primary outcome is a Composite measure of treatment failure, death, loss-to-follow-up and recurrence of tuberculosis disease. Treatment failure, death, loss-to-follow-up are assessed from the tuberculosis treatment register. Recurrence will be assessed through culture of sputum sample collected 6 months post treatment completion and patient self-report of restarting TB treatment by 12 months from enrolment. DALYs for a specific cause are calculated as the sum of the years of life lost due to premature mortality (YLLs) from that cause and the years of years of healthy life lost due to disability (YLDs) for people living in states of less than good health resulting from the specific cause.   The YLLs for a cause are calculated as the number of cause-specific deaths multiplied by a loss function specifying the years lost for deaths as a function of the age at which death occurs.  The trial outcomes are binary, but when converted to DALYs become a continuous outcome measure.

In addition to the health outcome, we will also report poverty cases averted (binary outcome measure) and catastrophic costs averted (binary outcome measure). Poverty cases averted are the proportion of individual income used to pay for accessing healthcare. If individual’s income reduces to below the national poverty line because of the out-of-pocket costs incurred in paying for healthcare, the individual is counted as a poverty case because of the cost of TB treatment. In the comparison between study arms therefore the poverty cases averted will be the differences between the proportion of poverty cases in the standard of care and the intervention arms of the study. While catastrophic costs averted are when the costs incurred by the individual exceeds 10% of their monthly income, the individual is said to have incurred catastrophic costs that would worsen household poverty over time.

# **Statistical analyses**

We will use Bayesian Hierarchical modelling with Markov Chain Monte Carlo (MCMC) to estimate differences in jointly modelled costs and outcomes between study arms (bivariate model), and to analyse associations between patient-level factors and costs and outcomes. Clusters are specified as random effects in the model. Due to the skewed nature of cost data, the response variables will be modelled using xero-inflated Poisson or lognormal regression models. We will use Stan in R for Bayesian inference for continuous variables, to obtain posterior simulations given the specified model and data using Markov Chain Monte Carlo sampling. Zero-inflated Poisson regression models don’t assume that zero and non-zero values come from the same data-generating process and models the cost variable as a mixture of Bernoulli and Poisson distribution. The convergence of the model will be assessed by looking at the Rhat values and plotting the MCMC chains and checking whether they are overlaying. We will also assess the conditional effect of the dependent variables on the independent variables. We will assess differences between real and simulated data by doing posterior predictive checking, comparing the replicated data under the fitted model to the observed data.

# **Economic analyses**

*Equity impact analysis*

The efficiency impact of the intervention will be assessed by using the values for costs and effectiveness calculated in the cost-effectiveness analysis to estimate the net health benefit of the intervention, using Equation 1.

|  | $NHB=\left( e_{i}-e_{c} \right)-(\frac{(c_{i}-c_{c})}{\gamma})$ | Equation 1 |
| --- | --- | --- |

Where in Equation 1, NHB refers to the *net health benefit* or the opportunity cost of lost health foregone elsewhere as a result of moving funding to pay for a new intervention; e*_i_* is the effectiveness of the intervention compared to the effectiveness of the comparator (e*_c_*) the current standard of care; c*_i_* refers to the cost of the intervention and c*_c_* to the cost of the comparator; and γ refers to the cost-effectiveness threshold.

The equity impact of the intervention will be assessed by estimating the next reduction in the health inequality index. The health inequality index will be calculated as the distribution of health outcomes by household socioeconomic position using the illness concentration index, using Equation 2.

|  | $C= \frac{2 cov (h_{i,} R_{i})}{h}$ | Equation 2 |
| --- | --- | --- |

The concentration index is derived from the concentration curve which plots the cumulative proportion of a health variable (here disability adjusted life years) against the cumulative proportion of the population ranked by household socioeconomic position. The concentration index is twice the area between the plotted concentration curve and the line of perfect equality.


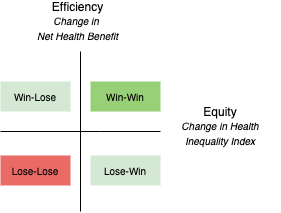


Figure S2. Equity-efficiency impact plane.

Investment in digital adherence technologies (DATs) are evaluated and presented on an equity-efficiency impact plane (see Figure S2). The plan presents the change in efficiency, measured in net health benefit and change in the health inequality index [3].

# **References**

1. Baio G. Bayesian models for cost-effectiveness analysis in the presence of structural zero costs. Stat Med. 2014;33: 1900–1913. doi:10.1002/sim.6074

2. Gomes M, Díaz-Ordaz K, Grieve R, Kenward MG. Multiple imputation methods for handling missing data in cost-effectiveness analyses that use data from hierarchical studies: an application to cluster randomized trials. Med Decis Mak Int J Soc Med Decis Mak. 2013;33: 1051–1063. doi:10.1177/0272989X13492203

3. Cookson R, Griffin S, Norheim OF, Culyer AJ. Distributional cost-effectiveness analysis: quantifying health equity impacts and trade-offs. Oxford; 2021.
